# Commentary: Teratogenic effects of the Zika virus and the role of the placenta

**DOI:** 10.3389/fcimb.2017.00062

**Published:** 2017-03-03

**Authors:** Shu Yuan, Qin Luo, Zhong-Wei Zhang, Zi-Lin Li

**Affiliations:** ^1^College of Resources, Sichuan Agricultural UniversityChengdu, China; ^2^School of Biomedical Sciences, Chengdu Medical CollegeChengdu, China; ^3^General Hospital of Lanzhou Military RegionLanzhou, China

**Keywords:** Zika virus, microcephaly, placental barrier, virus transfer, treatment time window, infection time

A widespread epidemic of Zika virus (ZIKV) infection was reported recently in South and Central America. The biggest concern to the ZIKV infection is the significant increase of microcephaly in the fetus born to the infected mother (Brasil et al., 2016; Johansson et al., 2016; Mlakar et al., 2016). The placenta acts as a barrier against the infection, due to its multiple unique structural, cellular and immune properties. However, the placenta may also play an important role in the virus transfer. One possibility is that the virus penetrates through the placental barrier. Zika virus could be packaged as a cargo for the placental exosome pathway at the endoplasmic reticulum in trophoblast cells. Then the secretory autophagy-pathway may cause secretion or expulsion of the viral particle rather than its degradation (Zhang et al., 2017). Alternatively, the infection to the placenta may induce several immune responses and lead to brain defects indirectly (Adibi et al., 2016). A recent study indicated that the ZIKV genome could be detected in the amniotic fluid (Calvet et al., 2016), and the complete genome of ZIKV can be also recovered from the fetal brain (Mlakar et al., 2016), all of which confirm that the virus can cross the placental barrier (Mysorekar and Diamond, 2016).

*In vitro* studies confirmed that ZIKV, but not the closely related dengue virus or West Nile virus, can infect key placental barrier cells efficiently (Richard et al., 2017). Although some reports indicated that Zika virus infection triggers apoptosis and vascular damage in the placenta, which may increase the permeability of the placenta (Aldo et al., 2016; Melo et al., 2016; Miner et al., 2016), two recent studies suggested that most trophoblast cells succeed in blocking ZIKV infection or only permitting a very low viral replication level (Bayer et al., 2016; Quicke et al., 2016). However, no other pathogenic flaviviruses cause congenital defects. Miner and colleagues' work mainly focused on placental and fetal cytological changes. They found that ZIKV infection induces trophoblast apoptosis and vascular damages in the placenta (Miner et al., 2016). El Costa et al. (2016) also demonstrated that ZIKV infects and damages tissue architecture of the fetal placenta, the maternal decidua basalis and umbilical cord. The structure of the placenta is complex, which is comprised of the maternal decidua and the fetal-derived compartments, including the labyrinth and junctional zones. Different types of trophoblast cells reside within all three layers. In the labyrinth-zone, fetal capillaries are lined by fetal blood vessel endothelium, which are segregated from maternal sinusoids by a layer of mono-nuclear trophoblasts and a syncytio-trophoblast bilayer (Adibi et al., 2016; Miner et al., 2016). However, only minimal trophoblast cell death was observed after the infection (Quicke et al., 2016) and thus the placental barrier might remain relatively intact. On the other hand, the placenta continues to produce trophoblast-derived interferons and other trophoblast-specific antiviral factors (Bayer et al., 2016; Quicke et al., 2016). Therefore, most ZIKV was blocked by trophoblast cells and the viral placental transfer might be a time-consuming process.

Placental transfer suggests that ZIKV must be transferred to the embryo at the early brain development stage (e.g., at the first trimester; Mlakar et al., 2016). However, at that time, the embryo has been shielded from the maternal blood, which nevertheless flows into the placenta only after 10 weeks of gestation (Adibi et al., 2016). Consistent with these structure changes, a tissue-level analysis in a case where the mother was infected at 7 weeks and miscarried at 12 weeks confirmed that the trophoblast was not infected by ZIKV at that period (Noronha et al., 2016). Thus, the virus may not reach the embryo before the first trimester.

However, for cases of ZIKV exposure in pregnancy, it is established that the greatest risk of microcephaly is in the pre-conception period and the first trimester (Johansson et al., 2016). In a case of late pregnancy, where infection occurred at 32 weeks, the virus was not detected in the fetal circulation, but it was detected in the placenta (Noronha et al., 2016). Similarly, in two cases of infection at 36 weeks, the virus was not detected in the infant's blood, although the newborns showed subependymal cysts (a kind of cerebral cysts) and lenticulostriate vasculopathy (lenticulostriate artery deformation) and they had normal neurological development for age as of the first postnatal month (Soares de Souza et al., 2016). Therefore, the viral placental transfer may take some time. Analysis of the clinical data (Brasil et al., 2016; Noronha et al., 2016; Soares de Souza et al., 2016) suggests that the virus may take about 5 weeks to reach the fetus for most cases (Table 1).

**Table 1:** **Time intervals between infections and abnormal findings of fetuses**.

**Fetus/Case No. (Reference)**	**Week of gestation at Infection**	**Week of gestation at the time of first abnormal finding of the fetus or the time of birth**	**Predicted time length of viral placental transfer (Weeks)**
Case 1 (Noronha et al., 2016)	7	Miscarried at 12 weeks (No fetal virus or neurological abnormality was found)	>5
Case 5 (Noronha et al., 2016)	32	Birth at 37 weeks (No fetal virus or neurological abnormality was found)	>5
Case 1 (Soares de Souza et al., 2016)	36	Birth at 38 weeks (subependymal cysts and lenticulostriate vasculopathy at birth, however has a normal neurological development later)	>2
Case 2 (Soares de Souza et al., 2016)	36	Birth at 39 weeks (subependymal cysts at birth, however has a normal neurological development later)	>3
Fetus 24 (Brasil et al., 2016)	12	29	17
Fetus 41 (Brasil et al., 2016)	12	24	12
Fetus 39 (Brasil et al., 2016)	21	30	9
Fetus 17 (Brasil et al., 2016)	22	26	>4
Fetus 12 (Brasil et al., 2016)	22	27	5
Fetus 10 (Brasil et al., 2016)	25	30	5
Fetus 36 (Brasil et al., 2016)	26	35	9
Fetus 38 (Brasil et al., 2016)	27	35	8
Fetus 2 (Brasil et al., 2016)	30	Birth at 34 weeks (Normal at birth)	>4
Fetus 53 (Brasil et al., 2016)	32	Still birth (Fetal death at 38 weeks)	6
Fetus 23 (Brasil et al., 2016)	35	Birth at 40 weeks (Electroencephalogram abnormalities)	5

As mentioned above, ZIKV infections may also induce acute inflammatory responses with up-regulation of interferons and cytokines and the indirect effects to the fetus (Adibi et al., 2016; Bayer et al., 2016; Mor, 2016; Quicke et al., 2016). Two case reports from Soares de Souza et al. (2016) indicated some congenital brain injuries in the blood ZIKV-negative fetuses (or the virus levels were under the limit of detection). The fetal brain injuries may be caused by the maternal infection indirectly, if the virus did not reach the fetus birth. Besides ZIKV, other flaviviruses also induce the cytokine storm. However, no relevance between any other flavivirus and microcephaly has been reported so far. Thus inflammatory responses may not play a major role in the incidence of lethal microcephaly.

We can postulate that if most ZIKV was cleared before it reaches the fetus, the incidence of microcephaly may be greatly decreased. The interferon therapy might help to clear the virus within 5 weeks. Alternatively, a combination of interferon with vaccines, newly-developed antiviral agents (e.g. *2*′*-C*-methylated nucleosides; Eyer et al., 2016) or exosome-specific inhibitors (Zhang et al., 2017) should be adopted for this therapeutic time window. The infection early in pregnancy may give the virus plenty of time to transfer through the placental barrier. And if the infection occurs early in pregnancy, the treatment window would be even longer, perhaps over 12 weeks.

## Author contributions

SY coordinated the writing and wrote this manuscript together with inputs form all other listed co-authors. SY and QL made equal contributions in finalizing this manuscript.

## Funding

This work was funded by the Preeminent Youth Fund of Sichuan Province (2015JQO045).

### Conflict of interest statement

The authors declare that the research was conducted in the absence of any commercial or financial relationships that could be construed as a potential conflict of interest.

## References

[B1] Adibi J. J. Marques E. T. Jr. Cartus A. Beigi R. H. (2016). Teratogenic effects of the Zika virus and the role of the placenta. Lancet 387, 1587–1590. 10.1016/S0140-6736(16)00650-4 26952548 PMC12626761

[B2] Aldo P. You Y. Szigeti K. Horvath T. L. Lindenbach B. Mor G. (2016). HSV-2 enhances ZIKV infection of the placenta and induces apoptosis in first-trimester trophoblast cells. Am. J. Reprod. Immunol. 76, 348–357. 10.1111/aji.12578 27613665

[B3] Bayer A. Lennemann N. J. Ouyang Y. Bramley J. C. Morosky S. Marques E. T. Jr.. (2016). Type III interferons produced by human placental trophoblasts confer protection against Zika virus infection. Cell Host Microbe 19, 705–712. 10.1016/j.chom.2016.03.008 27066743 PMC4866896

[B4] Brasil P. Pereira J. P. Jr. Moreira M. E. Ribeiro Nogueira R. M. Damasceno L. Wakimoto M.. (2016). Zika virus infection in pregnant women in Rio de Janeiro. N. Engl. J. Med. 375, 2321–2334. 10.1056/NEJMoa1602412 26943629 PMC5323261

[B5] Calvet G. Aguiar R. S. Melo A. S. Sampaio S. A. de Filippis I. Fabri A.. (2016). Detection and sequencing of Zika virus from amniotic fluid of fetuses with microcephaly in Brazil: a case study. Lancet Infect. Dis. 16, 653–660. 10.1016/S1473-3099(16)00095-5 26897108

[B6] El Costa H. Gouilly J. Mansuy J. M. Chen Q. Levy C. Cartron G.. (2016). ZIKA virus reveals broad tissue and cell tropism during the first trimester of pregnancy. Sci. Rep. 6, 35296. 10.1038/srep35296 27759009 PMC5069472

[B7] Eyer L. Nencka R. Huvarová I. Palus M. Joao Alves M. Gould E. A.. (2016). Nucleoside Inhibitors of Zika Virus. J. Infect. Dis. 214, 707–711. 10.1093/infdis/jiw226 27234417

[B8] Johansson M. A. Mier-y-Teran-Romero L. Reefhuis J. Gilboa S. M. Hills S. L. (2016). Zika and the risk of microcephaly. N. Engl. J. Med. 375, 1–4. 10.1056/NEJMp1605367 27222919 PMC4945401

[B9] Melo A. S. Aguiar R. S. Amorim M. M. Arruda M. B. Melo F. O. Ribeiro S. T.. (2016). Congenital Zika virus infection: beyond neonatal microcephaly. JAMA Neurol. 73, 1407–1416. 10.1001/jamaneurol.2016.3720 27695855

[B10] Miner J. J. Cao B. Govero J. Smith A. M. Fernandez E. Cabrera O. H.. (2016). Zika virus infection during pregnancy in mice causes placental damage and fetal demise. Cell 165, 1081–1091. 10.1016/j.cell.2016.05.008 27180225 PMC4874881

[B11] Mlakar J. Korva M. Tul N. Popović M. Poljšak-Prijatelj M. Mraz J.. (2016). Zika virus associated with microcephaly. N. Engl. J. Med. 374, 951–958. 10.1056/NEJMoa1600651 26862926

[B12] Mor G. (2016). Placental inflammatory response to Zika virus may affect fetal brain development. Am. J. Reprod. Immunol. 75, 421–422. 10.1111/aji.12505 26892436

[B13] Mysorekar I. U. Diamond M. S. (2016). Modeling Zika virus infection in pregnancy. N. Engl. J. Med. 375, 481–484. 10.1056/NEJMcibr1605445 27433842

[B14] Noronha L. D. Zanluca C. Azevedo M. L. Luz K. G. Santos C. N. (2016). Zika virus damages the human placental barrier and presents marked fetal neurotropism. Mem. Inst. Oswaldo Cruz 111, 287–293. 10.1590/0074-02760160085 27143490 PMC4878297

[B15] Quicke K. M. Bowen J. R. Johnson E. L. McDonald C. E. Ma H. O'Neal J. T.. (2016). Zika virus infects human placental macrophages. Cell Host Microbe 20, 83–90. 10.1016/j.chom.2016.05.015 27247001 PMC5166429

[B16] Richard A. S. Shim B. S. Kwon Y. C. Zhang R. Otsuka Y. Schmitt K.. (2017). AXL-dependent infection of human fetal endothelial cells distinguishes Zika virus from other pathogenic flaviviruses. Proc. Natl. Acad. Sci. U.S.A. 114, 2024–2029. 10.1073/pnas.1620558114 28167751 PMC5338370

[B17] Soares de Souza A. Moraes Dias C. Braga F. D. Terzian A. C. Estofolete C. F. Oliani A. H.. (2016). Fetal infection by Zika virus in the third trimester: report of 2 cases. Clin. Infect. Dis. 63, 1622–1625. 10.1093/cid/ciw613 27601223

[B18] Zhang Z. W. Li Z. L. Yuan S. (2017). The role of secretory autophagy in Zika virus transfer through the placental barrier. Front. Cell. Infect. Microbiol. 6:206. 10.3389/fcimb.2016.00206 28119857 PMC5220013

